# Knowing Your Beans in Parkinson's Disease: A Critical Assessment of Current Knowledge about Different Beans and Their Compounds in the Treatment of Parkinson's Disease and in Animal Models

**DOI:** 10.1155/2019/1349509

**Published:** 2019-10-30

**Authors:** Michel Rijntjes

**Affiliations:** Dept. of Neurology, Medical Center, University of Freiburg, Freiburg, Germany

## Abstract

This review contains a critical appraisal of current knowledge about the use of beans in both animal models and patients with Parkinson's disease (PD). The potential beneficial effects of beans in PD are increasingly being touted, not only in scientific journals but also by the lay media. While there is a long tradition in Ayurvedic medicine of prescribing extracts from *Mucuna pruriens* (MP), whose seeds contain 5% L-3,4-dihydroxyphenylalanin (L-DOPA), many other beans also contain L-DOPA (broad beans, common beans, and soybeans) or have other ingredients (coffee and cocoa) that may benefit PD patients. Indeed, bean-derived compounds can elicit neuroprotective effects in animal models of PD, while several studies in human PD patients have shown that motor performance can improve after ingestion of bean extracts. However, there are several arguments countering the view that beans serve as a natural therapy for PD: (i) the results from animal PD models are not necessarily directly applicable to humans; (ii) beans have many bioactive ingredients, some of which can be harmful in large doses; (iii) studies in human PD patients are scarce and only report on the effects of single doses or the administration of bean extract over short periods of time; and (iv) no data on long-term efficacy or side effects of bean therapy are available. Therefore, reservations about the use of beans as a “natural” therapy for PD seem to be justified.

## 1. Introduction

A physician taking care of a patient suffering from PD will eventually be asked whether it is advisable to take a preparation from the plant *Mucuna pruriens* (MP), whose seeds contain 5% L-DOPA [1]. Ayurvedic medicine has a long tradition of treating PD with MP, and many patients, especially in western countries, prefer to take “natural” remedies instead of pharmaceuticals. Moreover, when physicians ask their PD patients directly, they may be surprised about the high number who reveal that they already have experience with this herbal medicine.

The term “bean” is traditionally used for any plant belonging to the family Fabaceae—to which *Mucuna*, broad beans, common beans, and soy belong—but over time has also come to include seeds from other plants such as coffee and cocoa [2]. Beans other than MP are repeatedly mentioned in scientific reports and patient forums as being potentially beneficial in PD, due to the fact that they contain either L-DOPA (broad beans, common beans, and soy) or other compounds (coffee and cocoa). Therefore, some background information on the medical history of these types of beans results from animal models, and their use in PD might be helpful not only for advising PD patients, but also for assessing scientific reports and addressing the sometimes speculative claims by the media.

## 2. Methods

The primary database was PubMed. Search terms were the names of the six beans dealt with in this review (both their botanical and common names) in combination with either the term “parkinson” or “L-DOPA.” For cacao beans, additional search combinations were the combination of “theobromine” combined with “parkinson.” For coffee beans, the combination was “caffeine” and “parkinson.” All original studies that were thus retrieved and dealt with human subjects (also case reports) till March 2019 were included. Animal experiments were included when compounds showed clinical improvement in neuroprotective properties or might shed light on biochemical processes. Some articles led to references in journals and books that are not listed in PubMed, like agricultural journals, but especially historical sources. In such cases, the original texts in journals, books, and publications from NGOs were searched for information about beans on their first use in human society, their geographical dissemination, their use in traditional medicine, the first chemical isolation, relevant biochemical properties, and knowledge about compounds isolated that might have an impact on neuroprotection and Parkinson's disease. References where the original text was not available for scrutiny were not included.

### 2.1. *Mucuna pruriens* (Velvet Bean)

*Mucuna pruriens* (MP) is an annual plant originating from South-East Asia [3]. The term “pruriens” refers to the extreme itchiness that occurs after contact with the hairy pods, provoked by the toxin mucunain [4]. As far as it is known, MP contains the highest amount of L-DOPA [1], which plants deploy as a biological defense mechanism. Roots excrete L-DOPA in large amounts, in *Mucuna* plantations up to 400 kg L-DOPA per hectare per year [5]. L-DOPA is toxic to parasitic insects [6] such as nematodes, some of which infect plant roots. L-DOPA also inhibits the growth of neighboring plants—at a strength 100-fold higher than glyphosate [7]—likely interfering with amino acid synthesis and cell wall lignification [8]. L-DOPA is also toxic to herbivore insects; focal damage to *Mucuna* leaves, which already contain 1% L-DOPA, leads to local upregulation of L-DOPA [9].

In rat [10–12] and macaque monkey [13, 14] models of PD, MP preparations induced improvements in motor skills and dyskinesia similar to those induced by equivalent doses of L-DOPA. Another study compared the effects of L-DOPA vs. MP extract using a Drosophila model of autosomal recessive PD in which flies carried a mutation in the PTEN-induced putative kinase 1 (PINK-1) gene. Drosophila that was fed MP had a significantly extended lifespan, showed a restored olfactory response, and improved climbing behavior compared to flies that consumed L-DOPA [15]. Interestingly, MP was also reported to have neuroprotective properties, with the rescue of both tyrosine hydroxylase expression and damaged mitochondria. Additional animal studies have shown that MP exerts protective effects on neuronal cells under different neurotoxic conditions [16–18].

*Mucuna* seeds are rich in protein and in their processed form serve as a food supplement in several regions in Africa and Asia [19]. Raw seeds are not considered safe for human consumption due to the high amount of toxic alkaloids such as L-DOPA, as well as the hallucinogen dimethyltryptamine [20]. The concentration of these components can only be reduced by roasting, drying, or cooking, whereby the cooking water needs to be discarded repeatedly [3, 19]. Accordingly, during the 1989 civil war in Mozambique, mass psychosis was reported in a remote area in which the inhabitants were forced to nourish themselves with *Mucuna* beans, but had insufficient amounts of water to boil them in [20]. Their nonnutritious contents also limit the use of untreated *Mucuna* seeds as animal feed, except for ruminating mammals, who can adapt to L-DOPA via a microbial-driven fermentation process [3].

Ayurveda (Sanskrit: “Knowledge of life”) is the traditional form of medicine practiced in India. It assumes three kinds of energy (“dosha”) in the human body [21]: kapha, pitta, and vata, which are balanced in a healthy person. Vata (associated with the element of air) is the energy of movement, and too little or too much vata leads to diseases associated with the nervous system. The first description of PD symptoms in Ayurvedic literature possibly appeared around 600 BCE [22]. The Sanskrit word for tremor is “kampa,” and in 300 CE, the Indian physician Charaka used the term “Kampavata” to describe a syndrome characterized by bradykinesia, tremor, gait disorder, depression, and dementia [21]. Charaka recommended *Mucuna* (Sanskrit: “Atmagupta”) for the treatment of Kampavata.

The content of L-DOPA in dried and roasted *Mucuna* seeds varies between 4 and 6% (seeds from closer to the equator have a higher L-DOPA concentration [23]), while boiling reduces the content to about 1.5% [24]. Ayurvedic scripture suggests dissolving them in cow's milk [22]. The L-DOPA concentration of MP preparations depends on the method of extraction (e.g., with alcohol or water) [23, 25], and the dosage of L-DOPA in commercially available capsules varies widely between 60 and 500 mg.

Several studies have investigated the effect of MP in PD patients. *Mucuna* does not contain a DOPA decarboxylase inhibitor (DDCI), and without DDCI, the bioavailability of L-DOPA in the central nervous system is about one-fifth when combined with carbidopa or benserazide [26, 27]. Nonetheless, after a first report in 1978 on 23 PD patients who were treated with MP with similar effect and better tolerance than L-DOPA/benserazide [28], an open study in 60 PD patients in Hoehn and Yahr stage I-IV (about half of them were L-DOPA naïve) showed that treatment with MP preparations over 12 weeks led to significant improvements in both UPDRS score and the Hoehn and Yahr stage [29], which led to the registration of a *Mucuna* preparation (Zandopa™) as a treatment for PD in India.

When tested in small groups of PD patients in an advanced stage with motor fluctuations and peak-of-dose dyskinesias, a single dose of MP powder led to improvements in UPDRS, similar to the effects elicited by an equivalent dose of synthetic L-DOPA/DDCI and a longer “on” time [30, 31]. Interestingly, in one of these investigations, the pharmacokinetic profile of plasma L-DOPA concentration suggested that *Mucuna* formulations have a higher bioavailability than a standard L-DOPA preparation combined with DDCI [30].

In a crossover study (*N* = 14), PD patients in an advanced stage with motor fluctuations and peak-of-dose dyskinesias receiving a powder extract of *Mucuna* dissolved in water showed similar motor improvement compared to an equivalent dose of L-DOPA/benserazide, but half of the patients had to discontinue MP prematurely because of nausea (*N* = 3) or worsening motor performance (*N* = 4). This worsening was probably due to the reduced number of doses of L-DOPA per day with reoccurrence of wearing-off. However, those who discontinued because of nausea reported a better tolerability of ingesting the supernatant component from a solution of MP powder and water, without a change in MP-induced efficacy [32].

### 2.2. *Vicia faba* (Broad Bean)

*Vicia faba* beans were first cultivated in Mesopotamia [33] and then became widespread across the world, mainly because they also grow well in frosty areas such as northern Europe. Furthermore, dried fava beans have better storage properties than grains, making them suitable for use during winter [34]. In medieval Europe, fava beans and, to a lesser extent, peas and lentils represented the most important sources of protein since meat was expensive and its consumption was not permitted for religious reasons during the approximately 200 so-called “lean days” of the year [35].

Fava beans contain 0.5% L-DOPA (0.07% when dried) [1]. It was *Vicia faba* from which L-DOPA was first isolated by Guggenheim in 1913, while the discovery of the enzyme DOPA decarboxylase by Holtz in 1938 revealed that L-DOPA is the precursor of dopamine [36].

Although *Vicia faba* preparations have not been tested in animal models of PD, there do exist several anecdotal reports on their effects in humans. Apaydin and colleagues described 3 PD patients (Hoehn and Yahr stage II) who had “on-off” fluctuations and were given 250 g of cooked fava beans twice a day [37]. After one week of treatment, the authors observed prolonged “on” periods with reduced dyskinesia and markedly reduced time in the “off” period, while one patient was able to substantially reduce his L-DOPA medication. Apaydin et al. also reported that patients who tried dried beans did not experience any clinical benefits. Another case report describes an otherwise healthy 73-year-old PD patient in Hoehn and Yahr stage III who was on 800 mg L-DOPA/carbidopa per day and only had slight fluctuations. Two hours after eating a copious portion of freshly harvested raw fava beans, the patient experienced sudden agitation, severe chorea, tachycardia, anxiety, and vomiting, with full recovery 48 hours later [38]. Two small studies with 6 PD patients each compared the effects of a fava bean serving (cooked or microwaved) versus the equivalent dose of L-DOPA/carbidopa and reported that both the beans and the pharmaceuticals led to an improvement in the Webster score and caused dyskinesia to a similar extent [39, 40].

### 2.3. *Phaseolus vulgaris* (Common Bean and Green Bean)

Genetic studies of *Phaseolus vulgaris* suggest that this bean originated in Mesoamerica and later became domesticated in the Andes [41]. However, because the Andean variety thrives well in colder climates, it became the most popular strain in Europe [42]. The Arawak Indians who cultivated *Phaseolus* beans were spread throughout the Amazon basin from the Andean zone to the Caribbean [43] and were the first indigenous people that Columbus encountered in 1492. Upon returning from his first voyage, Columbus took some of the native people (most probably by force), birds, and plants back with him to show the King and Queen of Spain. Among the plants were *Phaseolus* beans, and he noted in his diary that they were “…fabas muy diversas de los nuestras…” [44]. From that point onwards, they became the most important food legume in the world for direct consumption [3]. According to the Food and Agriculture Organization of the United Nations (FAO), green beans are practically the “perfect food” because they are so rich in nutrients, acting as a source of protein, vitamins, minerals, fiber, complex carbohydrates, and iron [45].

*Phaseolus vulgaris* contains 0.25% L-DOPA [1]. One study in which rats were induced into a cataleptic state by chlorpromazine or haloperidol reported that treatment with a methanol extract of *Phaseolus* beans led to an improvement on a cataleptic score [46]. Despite the absence of reports on the use of *Phaseolus* in human PD patients, *Phaseolus* is being viewed as a potential therapeutic means in PD, and research efforts are being directed towards identifying the best cultivating conditions under which the amount of L-DOPA in *Phaseolus* can be increased [47].

### 2.4. *Glycine max* (Soybean)

Soybeans are native to East Asia and contain 0.02% L-DOPA [1]. Like *Vicia faba* and *Phaseolus*, they are rich in protein, vitamins, minerals, and fiber. The earliest mention of its domestication stems from China, and according to Chinese mythology, at around 2800 BCE, Emperor Shennong taught his people about the practice of agriculture and the use of herbal medicines, proclaiming that soy was one of five sacred plants [48].

Like in India, PD was probably already known in ancient China. In the Chinese medical classic the “Yellow Emperor's Internal Classic Plain Questions,” written in the 5^th^ century BCE, the following description of a disease is given: “A person appears with crouching of the head and with staring eyes, bending the trunk with shoulders drooped, with difficulty turning and rocking the low back, inability of the knees to flex and extend, with the back bowed, failure to stand for long periods, and tremor while walking” (cited from [49]).

In traditional Chinese medicine (TCM), diseases are caused by five different outside influences, namely (associated element): wind (wood), cold (water), heat (fire), dampness (earth), and dryness (metal) [50], and tremor and stiffness are thought to result from the influence of wind [49]. Since 300 BCE, soybeans have been used as herbal medicine for diarrhea and coughing [51], while several other herbs and plants are recommended for alleviating the symptoms of tremor and stiffness [49]. However, there is no evidence to suggest that soybeans were ever used as a therapy for a disease resembling PD.

There are a few reports on the neuroprotective effect of soybeans in animal models, one of them in toxin-induced parkinsonism [52]. A crossover design study in humans compared the effect of a single dose of 100 mg L-DOPA/25 mg carbidopa in 7 Parkinson patients, with or without the addition of 11 g of soybeans (from Hokkaido, roasted for 15 min at 150°C and then pulverized) [53]. After 3 hours, MDS-UPDRS III did not differ between treatment groups, but the addition of soybeans led to significantly less dyskinesias on the modified abnormal involuntary movement score (mAIMS) and a longer mean “on” period of 75 min, the latter assessed using a self-rating scale. The higher 3-O-methyldopa (3-OMD) and lower homovanillic acid (HVA) serum levels following supplementation with soybeans could be explained by an inhibitory effect on catechol-O-methyltransferase (COMT), suggesting that soybeans contain additional components that increase the bioavailability of L-DOPA. Soybean is a source of genistein [54], which was shown to inhibit peripheral DOPA decarboxylase activity [55]. This activity may influence the bioavailability of levodopa, though the daily amount needed to achieve an effective concentration is not known.

### 2.5. *Coffea* (Coffee Bean)

The coffee plant originates in Ethiopia, where coffee beans mixed with fat were traditionally used by wandering tribes for sustenance [56]. Coffee beans were known to the Persian physician Avicenna around the year 1000, who pointed out its invigorating properties, but for reasons unknown, it was only from the 16^th^ century onwards that brewed coffee became a worldwide popular drink, thanks to Arab traders [56]. Caffeine is part of the defense mechanism of the coffee plant, being toxic to herbivore insects. It was first isolated by the chemist Runge in 1819, after Goethe encouraged him to find the active ingredient of coffee beans [57].

Many studies have shown a dose-related inverse relationship between coffee consumption and the risk of developing PD (e.g., [58, 59]). This association is usually attributed to the effect of caffeine: serum levels of caffeine are reduced in patients in early stage PD compared to age-matched controls, irrespective of their actual coffee consumption [60]. Based on the results of animal model experiments, the proposed mechanisms underlying the neuroprotective and anti-inflammatory effects of caffeine include the following: (i) acting as an antagonist of the adenosine receptor A2a [61, 62]; (ii) increasing the plasma levels of the neurotrophic factor G-CSF [63]; and (iii) directly interfering with a-synuclein folding [64]. The dietary fiber derived from coffee beans has been reported to increase the amount of fecal *Prevotella* bacteria [65], of which a low concentration in the human microbiome has been linked to PD [65, 66].

An open study in PD patients showed that ingestion of 200 mg caffeine daily (equivalent to two cups of coffee) could improve the UPDRS score by an average of 5 points [67]. However, chronic coffee intake during the early stages of PD does not correlate with radionuclide uptake in DAT-SPECT [68], while in a double-blind, placebo-controlled, randomized study over 1.5 years, a daily intake of 200 mg caffeine had no effect on a cohort of 60 PD patients [69].

One explanation for these apparently contradictive observations is the possibility that the correlation between the PD risk and coffee consumption (as well as smoking) might be subject to the “chicken or the egg” conundrum: because a parkinsonian personality is characterized by low sensation-seeking traits, people who are at risk of developing PD have less tendency to use stimulating substances such as caffeine (or nicotine) [70].

### 2.6. *Theobroma cacao* (Cocoa Bean)

The cacao tree is native to South America and was widely cultivated before the arrival of the conquistadores. For the Aztecs, cocoa beans imported from the more southern Maya regions were considered a luxury. They were used as currency, and at the court of Montezuma, a drink (“chocolatl”) was prepared from roasted cocoa beans mixed with vanilla and chili [71]. Cocoa beans were named *Theobroma* (“food of the gods”) by Linnaeus. Its primary alkaloid with a concentration of 1% is the xanthine theobromine, which was first isolated from cocoa by the chemist Woskresensky in 1842 [72] and has a mildly stimulating effect on humans. Most animals metabolize theobromine at a much slower rate to humans, and even small quantities (1 g/kg) of dark chocolate are poisonous to dogs, inducing vomiting, increased urine output, progressing to epileptic seizures, and arrhythmia [73].

Parkinson patients have an increased chocolate consumption rate compared to healthy controls [74]. Several components of cocoa that cross the blood-brain barrier and may improve mood have been implicated, especially xanthines such as theobromine and caffeine, as well as phenylethylamine. Phenylethylamine is not only a precursor of dopamine, but it also stimulates the release of norepinephrine and dopamine into the synaptic cleft [75], leading to a feeling of well-being. Theobromine is an adenosine receptor antagonist like caffeine and appears to have additional properties, such as reducing cellular oxidative stress and regulating gene expression [76]. Also, it induces the expression of brain-derived neurotrophic factor, which is associated with improved motor learning in a mouse model of PD [77].

In healthy human subjects, dark chocolate improves mood and general cognitive function whereas white chocolate (devoid of cocoa) does not [78]. However, when the effect of theobromine is directly compared to that of caffeine in healthy subjects, the positive effect on mood appears mainly attributable to caffeine [79, 80], even when taking into consideration the fact that the liver metabolizes caffeine to 12% theobromine in humans [81]. Furthermore, in a crossover design study of moderately affected PD patients, a single 200 mg dose of dark chocolate with 80% cocoa did not differ from 200 mg of white chocolate in terms of its effects on the UPDRS motor score, Global Clinical Impression Score, or Beck's Depression Inventory [82].

Clovamide (*N*-caffeoyl-L-DOPA), a conjugate of L-DOPA, was first isolated from red clover (*Trifolium pratense*) [83], which belongs to the bean family (Fabaceae), and later extracted from cocoa beans [84]. There are no reports of clovamide itself having a dopaminergic action, nor that it can be metabolized into its components in humans. Clovamide has neuroprotective properties in *in vitro* models of oxidative stress, excitotoxicity damage, and ischemia/reperfusion [85]. Several analogues of clovamide are now being investigated, both for their effectiveness against oxidative stress [86] and for their anti-inflammatory properties [87], with the aim of leading to a neuroprotective treatment in PD.

## 3. Critical Appraisal and Conclusion

The types of beans described in this overview all appear to have neuroprotective properties in animal models of PD, some of them being associated with improved motor performance. These effects are seen after the administration of either a bean extract or an individual compound. However, some important facts need to be kept in mind regarding animal models of PD. Firstly, the neuroprotective effects of individual compounds in animals are sometimes only observed in doses that would be toxic to humans [61]. Secondly, animal models usually focus on basal ganglia pathology and motor performance, whereas in humans, deposits of a-synuclein—the signature protein of PD—are found in many other parts of the central and peripheral nervous system, often long before the characteristic motor symptoms become apparent [88]. Thirdly, animals do not suffer from PD in the wild. Animal models of PD are based on genetic mutations or toxin-induced parkinsonism, and therefore, the findings are not necessarily applicable to a slow-progressing neurodegenerative disease in humans.

Extracts from most beans containing L-DOPA can improve motor symptoms in PD patients, sometimes with reduced dyskinesia. However, most studies only report the results after administration of a single dose of extracts in small groups of patients, or as case reports, and data on bean extracts given over periods longer than a few months are not available. An international, multicenter RCT on the noninferiority of *Mucuna* powder versus commercial levodopa/benserazide 200/50 mg tablets for long-term efficacy and safety is currently ongoing in newly diagnosed PD patients in the early stage of disease (Pan African Clinical Trials Registry, number 201611001882367).

It has to be taken into account that all beans have many ingredients that may be biologically active. For example, although cohort studies show that moderate consumption of coffee (3–5 cups per day) is associated with the decreased risk for developing several chronic ailments such as cerebrovascular disease [89], diabetes [90], and dementia [91], the effects of coffee and other beans might not be due to one ingredient alone (e.g., caffeine and L-DOPA), but rather to the synergistic actions between any other of the hundreds of different endogenous compounds (e.g., [15, 92]).

Individual compounds can also have side effects. While some are merely inconvenient, like the flatulence after *Phaseolus* consumption caused by fermentation of galactooligosaccharides [93], others can be harmful. The psychotropic effects of alkaloids in *Mucuna pruriens* were mentioned before, and consumption of a few raw kidney beans, a variety of *Phaseolus vulgaris*, leads to vomiting and diarrhea, due to the toxin phytohaemagglutinin [94]. The most consequential example is favism in individuals with glucose-6-phosphate dehydrogenase (G6PD) deficiency, which affects about 400 million people worldwide [95]. *Vicia faba* contains considerable amounts (2% of dry weight) of the *β*-glucosides vicine and convicine, whose metabolites produce highly reactive oxygen species that cannot be detoxified within G6PD-deficient red blood cells, leading to hemolysis [96].

In addition to PD, Ayurvedic medicine recommends MP for many other affections such as snakebites, worm infections, diabetes, epilepsy, psoriasis, and depression, and as an aphrodisiac [97]. These last two indications could be attributed to the high concentration of L-DOPA. However, *Mucuna* seeds also contain many other compounds [3, 25], among them the degradation products of L-DOPA, which include quinones, other oxygen radicals, and several more reactive oxygen species [98]. Quinones might be the reason for the antimicrobial and antiparasitic use of MP in traditional medicine, but like oxygen radicals and other reactive oxygen species, they might play a role themselves in the development of PD [99].

MP was suggested as an alternative source of levodopa for PD patients who cannot afford therapy with commercial levodopa preparations [31]. However, because the long-term effects of the other compounds are unknown (with many of them not even being identified yet), others suggest a large amount of restraint [98]. Moreover, levodopa-induced dyskinesias develop in up to 80% of PD patients, depending on dosage and duration [100]. Regular ingestion of MP could accelerate the incidence of dyskinesia, and long-term data on MP in PD are completely lacking. Therefore, any study on the effects and tolerability of *Mucuna* in PD should be accompanied by measurements of plasma concentrations for an accurate calculation of L-DOPA intake.

Still, in low-income countries in which access to pharmaceutical L-DOPA preparations is severely limited, *Mucuna* might offer a potential alternative source of L-DOPA. In these cases, the intake of *Mucuna* preparations should be under supervision of physicians trained on its potential side effects and how to adjust the daily dosage, especially since the dosage in commercially available *Mucuna* capsules has a very wide range. However, patients taking MP preparations should be advised against concomitant intake of pharmaceutical preparations of L-DOPA, since the presence of the decarboxylase inhibitor might potentiate side effects.

In conclusion, until more information is available, reservations about the use of beans as a “natural” therapy for PD seem to be justified for the time being.
